# Invertebrate extracellular phagocyte traps show that chromatin is an ancient defence weapon

**DOI:** 10.1038/ncomms5627

**Published:** 2014-08-13

**Authors:** Calum T. Robb, Elisabeth A. Dyrynda, Robert D. Gray, Adriano G. Rossi, Valerie J. Smith

**Affiliations:** 1 Centre for Marine Biodiversity and Biotechnology, School of Life Sciences, Heriot Watt University, Edinburgh, Scotland EH14 4AS, UK; 2 Centre for Inflammation Research, Queen’s Medical Research Institute, University of Edinburgh, Edinburgh, Scotland EH16 4TJ, UK; 3 Scottish Oceans Institute, School of Biology, University of St Andrews, St Andrews, Scotland KY16 8LB, UK

## Abstract

Controlled release of chromatin from the nuclei of inflammatory cells is a process that entraps and kills microorganisms in the extracellular environment. Now termed ETosis, it is important for innate immunity in vertebrates. Paradoxically, however, in mammals, it can also contribute to certain pathologies. Here we show that ETosis occurs in several invertebrate species, including, remarkably, an acoelomate. Our findings reveal that the phenomenon is primordial and predates the evolution of the coelom. In invertebrates, the released chromatin participates in defence not only by ensnaring microorganisms and externalizing antibacterial histones together with other haemocyte-derived defence factors, but crucially, also provides the scaffold on which intact haemocytes assemble during encapsulation; a response that sequesters and kills potential pathogens infecting the body cavity. This insight into the early origin of ETosis identifies it as a very ancient process that helps explain some of its detrimental effects in mammals.

## Abstract (web summary)

The process of controlled chromatin release from the nuclei of inflammatory cells to entrap and kill bacteria, termed ETosis, is important in innate immunity in vertebrates. Here the authors demonstrate that ETosis, mediated by hematocytes, also contributes to defence mechanisms in invertebrates.

## Graphical abstract

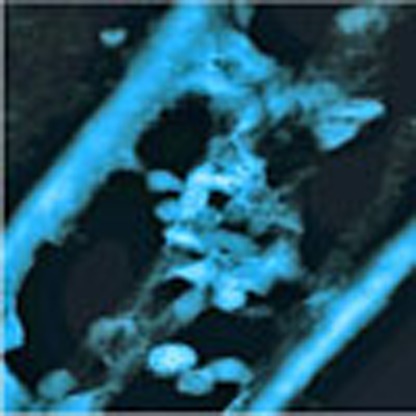

ETosis was first discovered in mammalian neutrophils^1^ and is now known to occur in a variety of mammalian innate immune cells^2^,^3^,^4^,^5^. The phenomenon entails the expulsion of chromatin from the nucleus via reactive oxygen species (ROS) involvement and can be induced by various agents such as the protein kinase C activator, phorbol myristate acetate (PMA), H_2_O_2_ from the respiratory burst, lipopolysaccharide (LPS) or bacteria^6^,^7^,^8^. The discharged chromatin forms complex meshes that ensnare and kill bacteria, fungi, viruses and other parasites^7^,^9^,^10^. The process is now widely regarded as an important part of the mammalian inflammatory repertoire, but has also been implicated in a number of disease states, such as thrombosis, mastitis, appendicitis, preeclampsia, arthritis and promotion of cancer metastasis^11^,^12^,^13^. Although mainly described in mammalian systems, ETosis also occurs in chicken heterophils^14^ and fish phagocytes^15^. However, the presence and role of ETosis in invertebrates has not been explored in detail, even though understanding the phylogeny of the process may help explain its varied effects in mammals.

As invertebrates depend on the innate activities of blood cells for defence and possess the key structures and protein homologues necessary to execute inflammation, we hypothesized that ETosis could be an important primordial defence strategy. For animals in lower taxa, especially those with an open circulatory system, we theorized that chromatin externalized by ETosis would represent a powerful way to contain infectious agents gaining entry into the body cavity and could play a role in the encapsulation of foreign materials by the haemocytes. For these studies, the shore crab, *Carcinus maenas*, was used as the main experimental animal because it possesses large populations of haemocytes that can be separated on Percoll and manipulated easily *in vitro*^16^. To investigate if ETosis occurs more widely across the invertebrates, comparative studies were also made on a distantly related protostome, the blue mussel, *Mytilus edulis*, and a cnidarian, specifically the sea anemone, *Actinia equina.* Mussel haemocytes are avidly phagocytic and display a strong respiratory burst^17^,^18^. Sea anemones, while not possessing a dedicated coelomic immune system, nevertheless, contain phagocytes within the mesoglea that are known to undergo a respiratory burst^19^ and express ROS-relevant genes^20^. Therefore, we performed a study to ascertain if immune cells from these invertebrates exhibit ETosis.

The findings reported here confirm that invertebrate defence cells are indeed capable of ETosis and at least in crab, the process contributes to encapsulation reactions mounted against non-self agents. That an acoelomate invertebrate also undergoes an ETotic-type response shows for the first time that chromatin release is an ancient and evolutionary conserved mechanism.

## Results

### Characterization of chromatin release from crab haemocytes

**Figure 1:**
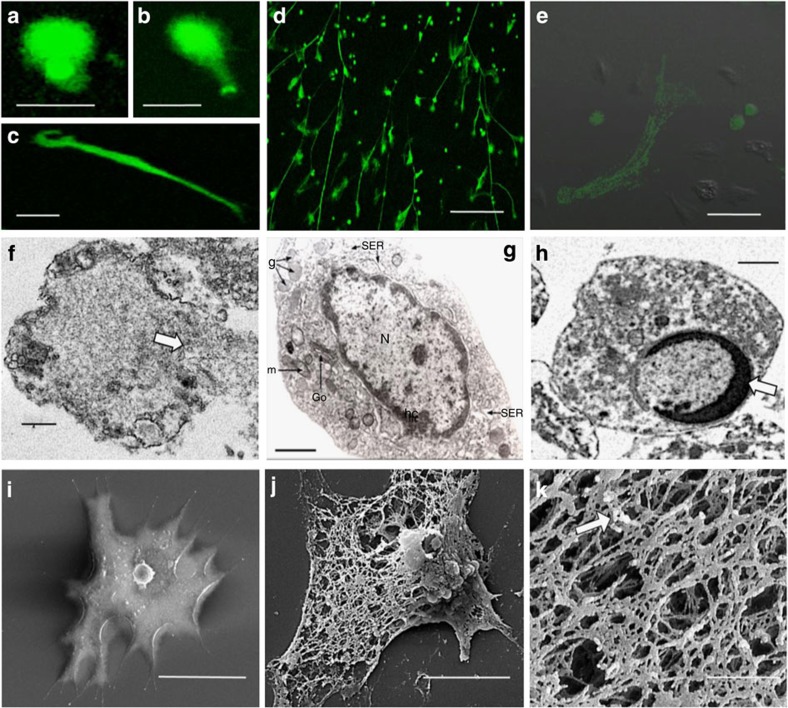
Chromatin discharge by separated HCs from *C. maenas in vitro*. (**a**–**e**) Unfixed HCs in monolayer cultures stained with Sytox Green. (**a**,**b**) Diffuse extracellular chromatin at 3 h, 1.0 μM PMA. (**c**) Spread extracellular chromatin at 24 h, 0.1 μM PMA. (**a**–**c**) Scale bar, 30 μm. (**d**) Extended extracellular chromatin strands (24 h, 0.1 μM PMA ). Scale bar, 300 μm. (**e**) Merged phase contrast and fluorescence images of an ETotic HC at 24 h (0.1 μM PMA). Also evident are viable cells and stained necrotic/late apoptotic cells with round stained nuclei. Scale bar, 50 μm. (**f**) TEM of a HC (24 h, 0.1 μM PMA) showing swelling of uncondensed chromatin, nuclear membrane breakdown and chromatin discharge from the cell at a breach point (arrow). (**g**) TEM of a control, unstimulated HC, N, nucleus with intact membrane and rim of condensed heterochromatin (hc) at the periphery. Go, Golgi body, g, granules, m, mitochondrion, SER, smooth endoplasmic reticulum. (**h**) TEM of an apoptotic HC showing an intact nuclear membrane with a thick, crescent shaped rim of condensed chromatin (arrow). (**f**–**h**) Scale bars, 1 μm. (**i**) SEM of control HC. Scale bar, 20 μm. (**j**) SEM of ETotic HC (24 h, 0.1 μM PMA). Scale bar, 5 μm. (**k**) SEM detail of extracellular chromatin released from a HC (24 h, 0.1 μM PMA). Arrow indicates granules studded on the extracellular mesh. Scale bar, 1 μm.

Hyaline cells (HCs) were utilized for initial experiments, as among the four types of haemocyte in *C. maenas*, they are the only ones known to be both phagocytic and able to undergo a respiratory burst^21^,^22^. We found that treating preformed monolayers of isolated populations of crab HCs with PMA resulted in expulsion of material staining positively with Sytox Green (Fig. 1a–e). This reagent binds DNA but only permeates cells that have lost membrane integrity and thus reveals dead cells or exposed nucleic acid. Ejection of chromatin usually occurs between 2 and 24 h with the extruded material forming diffuse (puffball-like) structures (Fig. 1a,b), spread (comet-like) structures (Fig. 1c) or extended strands that interlink across cells (Fig. 1d,e), similar to the morphology of NETs produced by mammalian neutrophils^23^. Maximal expulsion of chromatin was achieved with 0.1 μM PMA over 24 h (Supplementary Fig. 1a,b), with 63.4±5.1% s.e.m. of the haemocytes showing the phenomenon under these conditions. By contrast, the proportion was <5% in the controls. Importantly, DNAse-1 completely dissolved the material released from the HCs, confirming that it is chromatin (Supplementary Movie 1). Transmission electron microscopy revealed that nuclear membrane breakdown precedes chromatin release (Fig. 1f), as in neutrophils^6^. Moreover, there is no peripheral condensation of chromatin at the nuclear membrane, in contrast to healthy cells (Fig. 1g) or apoptotic cells (Fig. 1h; Supplementary Fig. 2a–d) Rather, scanning electron microscopy (SEM) showed that, unlike untreated haemocytes (Fig. 1i), extruded DNA forms a large extracellular complex mesh of strands (Fig. 1j) studded with small granules resembling globules (Fig. 1k). Thus expelled chromatin from crab haemocytes has a similar structure to mammalian NETs, although the smooth domains are *ca.* 26.0 nm (±1.75 nm s.e.m.) in diameter while the globular domains are 47.8 nm (±3.4 nm s.e.m.) in diameter, both of which are slightly larger than those previously determined for neutrophils^1^. Chromatin is similar in its dimensions across all animal taxa, but, at the ultrastructural level, differences in measurements may be introduced due to processing, type of microscopy and the analysis software used.

**Figure 2:**
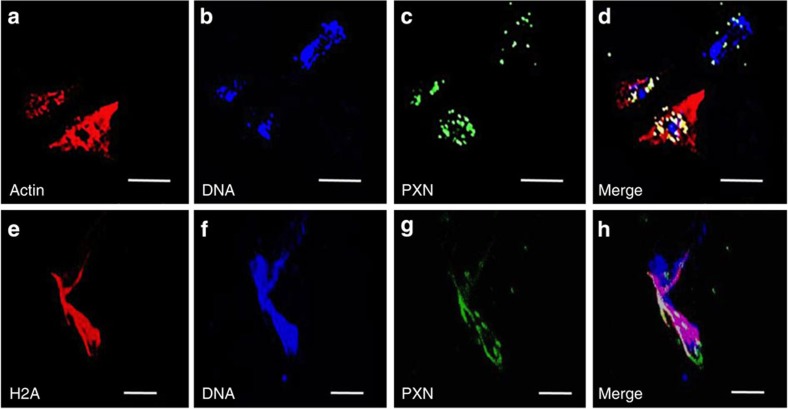
Immunocytochemical analysis of chromatin released from HCs of *C. maenas in vitro.* (**a**–**d**) Localization of actin, DNA or PXN in an ETotic haemocyte (top right) and two non-ETotic haemocytes (bottom left), 24 h incubation with 0.1 μM PMA. (**a**) Actin shown by rhodamine phalloidin. (**b**) Visualization of DNA by Draq 5. (**c**) Localization of PXN, by anti-myeloperoxidase (MPO) antibody. (**d**) Merge of **a**–**c**. All scale bars, 25 μm. (**e**–**h**) Localization of histone H2A, DNA or PXN in a different ETotic cell. (**e**) Localization of H2A. (**f**) Visualization of DNA by Draq 5. (**g**) Localization of PXN, by anti-MPO antibody. (**h**) Merge of **e**–**g**. Note H2A co-localizes with extracellular DNA and PXN. All scale bars, 20 μm.

Immunocytochemical analyses to evaluate the decoration of the disgorged chromatin from HCs confirmed that it becomes associated with a protein recognized by an anti-myeloperoxidase antibody (Fig. 2a–d). This protein, peroxinectin (PXN), is a crustacean homologue of myeloperoxidase that is expressed by haemocytes involved in cell-to-cell adhesion^24^,^25^. In the present study, it was released from the perinuclear region of the HCs, as the antibody did not co-localize with the nucleus in intact cells (Fig. 2d). In addition, actin did not co-localize with the extracellular chromatin released from crab HCs (Fig. 2b,d) as was observed in NETs released from fathead minnow^15^. Crucially, we also demonstrate that histone H2A is liberated from the nuclei of the HCs to the extracellular environment (Fig. 2e–h). Non-specific staining was not apparent in any controls. Histones are highly conserved nuclear proteins with potent antibacterial properties^26^. ETosis is the only known mechanism by which they could be exposed to infective agents. Indeed decoration of chromatin NETs by histones is one of the characteristic features of the phenomenon for mammals^6^.

**Figure 3:**
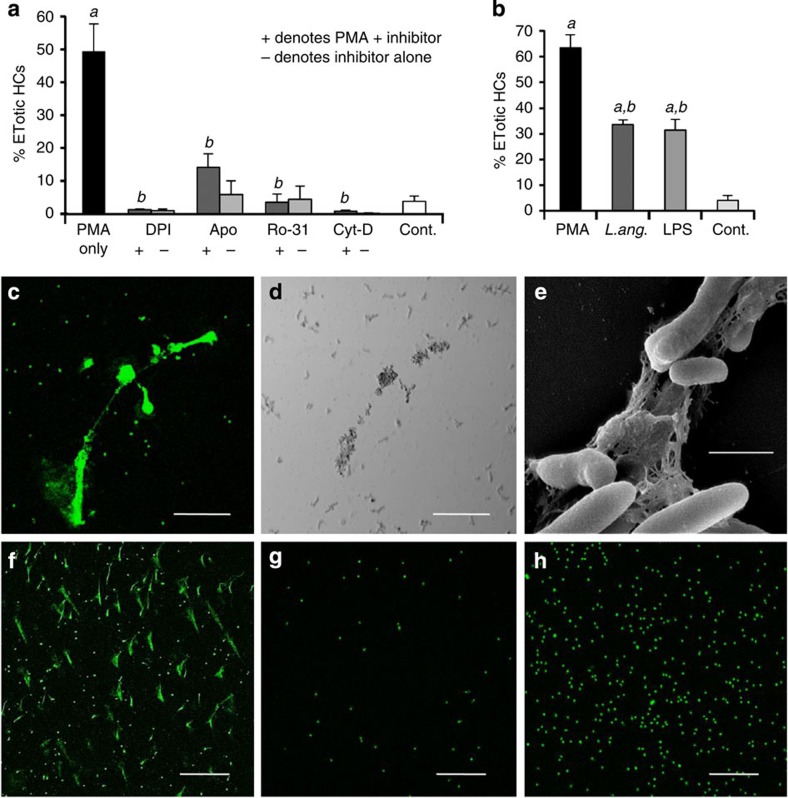
Chromatin release in cellular defences of *C. maenas in vitro.* (**a**) Percentage of *C. maenas* HCs following PMA treatment (24 h, 0.1 μM) or PMA plus the inhibitors DPI (2 μM), apocynin (Apo) (50 μM), Ro-31-8220 (Ro-31) (1 μM), cytochalasin D (Cyt-D) (10 μM) or left untreated (Cont.). Values are mean±s.e.m., *n*=3. Significant differences between PMA treatments and untreated controls denoted by *a*; Significant differences between PMA and PMA +/− inhibitor denoted by *b*. All *P* values are <0.001, one-way analysis of variance (ANOVA) with Student–Newman–Keuls (SNK) *post hoc* test. (**b**) Percentage of ETotic *C. maenas* HCs following treatment with PMA (0.1 μM), live *L. anguillarum* (3 × 10^5^ ml^−1^) or LPS (0.1 μg ml^−1^) compared with controls. All cultures incubated at 10 °C for 24 h. Values are mean±s.e.m., *n*=3. Significant differences between the control and PMA, LPS or *L. anguillarum* are indicated by *a*. Significant differences between PMA and LPS or *L. anguillarum* are indicated by *b*. All *P* values are <0.001, one-way ANOVA with SNK *post hoc* test. (**c**) Unfixed, ETotic HCs (24 h, *L. anguillarum* (3 × 10^4^ ml^−1^) stained with Sytox Green. Stained small dots in background are bacteria. (**d**) Phase contrast view of (**c**). (**c**,**d**) Scale bar, 75 μm. (**e**) SEM of chromatin released from an HC, trapping *L. anguillarum*. Scale bar, 1 μm. (**f**–**h**) Effect of PMA (24 h, 0.1 μM) on other haemocyte populations, stained with Sytox Green. (**f**) ETotic SGCs. (**g**) Non-ETotic prohaemocytes. (**h**) Non-ETotic GCs. (**f**–**h**) Scale bars, 300 μm.

The release of the extracellular chromatin by crab HCs was significantly reduced by diphenylene iodonium (DPI), apocynin, Ro-31-8220 and cytochalasin D (all *P*<0.001) (Fig. 3a). DPI inhibits flavoprotein oxidoreductases (including nicotinamide adenine dinucleotide phosphate (NADPH) oxidase) while apocynin inhibits the assembly of NADPH oxidase^27^. Ro-31-8220 is a pan protein kinase C inhibitor^28^ that inhibits generation of ROS upstream of NADPH oxidase activation whereas cytochalasin D prevents actin polymerization^29^. These agents also inhibit ETosis by mammalian neutrophils^6^,^8^,^30^,^31^, because NADPH oxidase and the cytoskeleton have crucial and overlapping roles in the process: NADPH oxidase is needed for decondensation of chromatin before its discharge, and the cytoskeleton is required for NADPH oxidase assembly^31^. Actin filamentation is also necessary for positioning the nucleus close to the plasma membrane and for rupture of the cell, hence the inhibitory effect of cytochalasin D, which also impairs histone citrullination^31^. Ro-31-8220 has been demonstrated to inhibit generation of ROS in human neutrophils^8^. It is therefore likely that chromatin release by crab HCs involves similar processes as those used by mammalian cells, so we conclude that the results taken together show that crab HCs are capable of undergoing vertebrate-type ETosis.

### ETosis in crustacean host defence

To evaluate the role of chromatin release in crab host defence, additional *in vitro* experiments were performed using LPS or *Listonella anguillarum*, a mild bacterial pathogen of decapods, as the initiator. Both were found to significantly stimulate chromatin expulsion from the HCs within 24 h (*P<*0.001 compared with control) with no significant difference in the percentage of ETotic cells between treatment with LPS and the bacterium (Fig. 3a,b). With *L. anguillarum*, the bacteria become closely associated with the chromatin strands (Fig. 3c–e), in a similar way to the entrapment of bacteria by ETotic neutrophils^6^. The proportion of HCs induced by LPS or bacteria to extrude chromatin was found to be 31.0% (±4.2% s.e.m.) and 33.6% (±1.9% s.e.m.), respectively, after 24 h culture (10 °C) (Fig. 3b). These levels are lower than that achieved with PMA but perhaps reflect a more realistic level of response to natural infection. In both cases, the frequency of the responses were reduced to <2% by pretreatment with DPI (*P*<0.001).

Of the three other haemocyte types in *C. maenas*, only the semi-granular cells (SGCs) exhibited chromatin release 24 h after PMA treatment (0.1 μM) *in vitro* (Fig. 3f). Neither the prohaemocytes (immature haemocytes) nor the granular cells (GCs) showed the response (Fig. 3g,h). Curiously, there was better survival of prohaemocytes (Fig. 3g) than all other haemocyte types, whereas GCs showed the poorest survival as revealed by the large number of positively stained, non-chromatin extruding nuclei (Fig. 3h). The proportion of SGCs externalizing chromatin at this time was 50.8% (±3.8% s.e.m.), with <5% in the controls. In crab, the SGCs are known to degranulate and die in the presence of bacteria or LPS^32^. From the present study, it appears that the cells die through ETosis, although it remains unclear if SGCs are capable of a NADPH oxidase-dependent respiratory burst^22^. SGCs and GCs do, however, cooperate with the HCs in the encapsulation of microorganisms *in vivo*^33^ and both show antimicrobial activity, in contrast to crab HCs, which do not^21^.

### Participation of ETosis in encapsulation reactions

**Figure 4:**
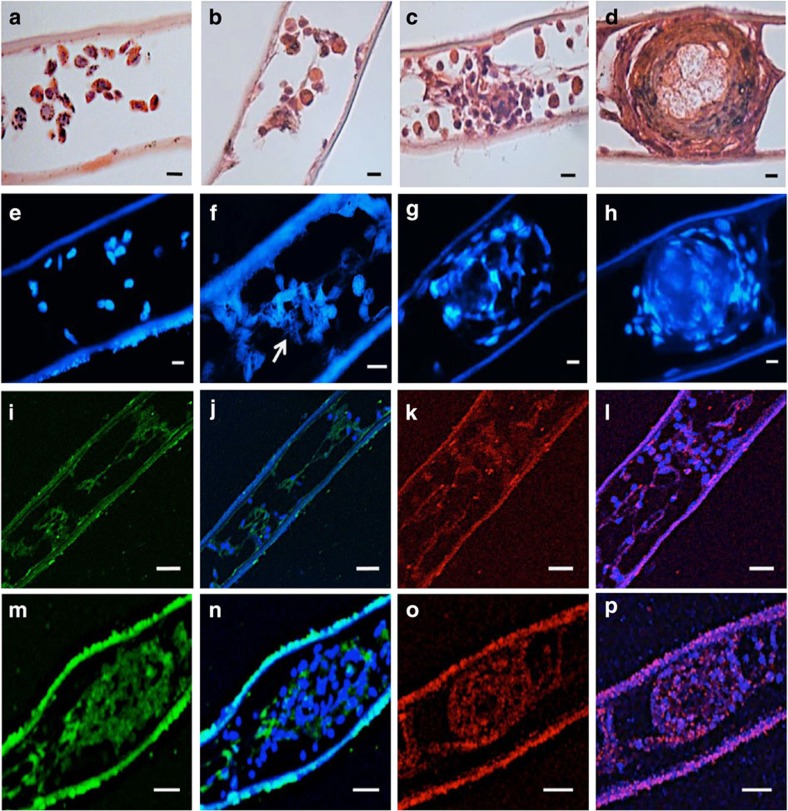
*In vivo* role of ETosis during host defence in *C. maenas.* (**a**–**d**) Haematoxylin and eosin-stained paraffin wax sections of gill lamellae at various incubation periods after 100 μl injection of LPS (20 μg ml^−1^) or saline. (**a**) Control, 24 h after injection of sterile saline. (**b**) Part of a gill lamella 1 h after LPS treatment. (**c**) Early capsule formed 3 h after LPS treatment. (**d**) Fully formed capsule 24 h after LPS injection. (**e**–**h**) 2-(4-amidinophenyl)-1H-indole-6-carboxamidine (DAPI)-stained paraffin wax sections of gill lamellae after 100 μl injection of LPS (20 μg ml^−1^) or saline. (**e**) Control, saline treatment (24 h). Epibionts are evident on the external (seawater) surface of the lower lamella surface and there is no haemocyte clumping or externalized chromatin. (**f**) 1 h post LPS treatment showing ETotic cells within a gill lamella. Arrow indicates likely externalized chromatin. (**g**) Haemocyte clump 3 h post LPS treatment. (**h**) Fully formed capsule 24 h post LPS treatment. (**i**–**l**) Immunohistochemical staining of haemocyte aggregations formed at 1 h post LPS treatment. (**i**) PXN visualization with anti-MPO antibody. (**j**) Merge of **i** with same section stained with TO-PRO-3 iodide to reveal DNA. (**k**) Visualization of H2A in a different area of lamella. (**l**) Merge of **k** with same section stained with TO-PRO-3 iodide. (**m**–**p**) Immunohistochemical staining of a haemocyte clump 3 h post LPS treatment. (**m**) Visualization of PXN with anti-MPO antibody. (**n**) Merge of **m** with same section stained with TO-PRO-3 iodide. Note the mostly extracellular location of PXN. (**o**) Visualization of H2A in a different clump. (**p**) Merge of **o** with same section stained with TO-PRO-3 iodide. H2A co-localization is pronounced and occurs throughout clump structure. All scale bars, 20 μm.

Experiments to test the role of ETosis in encapsulation *in vivo* confirmed that 24 h following LPS injection, capsules formed and lodged within the gill lamellae (Fig. 4a–d) in a similar way to that reported previously with bacteria or β-1,3 glucans^33^,^34^. There is progression of haemocyte aggregation from 1 h post treatment, when intact haemocytes begin to associate (Fig. 4b), through coalescence into loose clumps at 3 h (Fig. 4c) and eventually development of fully formed, compact, structures after 24 h (Fig. 4d). Staining with 2-(4-amidinophenyl)-1H-indole-6-carboxamidine (DAPI) revealed that some haemocytes release chromatin within the first hour (Fig. 4f), but by 3 h, extracellular chromatin is much more in evidence, predominantly in the peripheral region of the clumps (Fig. 4g). By 24 h, chromatin is conspicuously diffused throughout the compacted cores of the capsules (Fig. 4h). Similar clumping was not apparent in the gills of the control crabs even after 24 h (Fig. 4a,e). Immunohistochemical analysis confirmed that at 1 h post LPS treatment, PXN is visible in the lumen of the gill filaments but is largely external to the cells (Fig. 4i,j). By contrast, some H2A does co-localize with extracellular nucleic acid (Fig. 4k,l). In the 3-h clumps, PXN is discernable within the clumps mainly in the outer regions and interspersed between intact cells (Fig. 4m,n). Co-localization of H2A with DNA, however, is more pronounced and occurs throughout the clump structure (Fig. 4o,p). Neither PXN nor H2A were seen extracellular to the haemocytes or the gill tissue of the sections from the controls. At 24 h, the capsules in the gills of experimental crabs were so densely compacted that visualization of individual elements was no longer possible. Autofluorescence was observed on the external surface (seawater side) of the gill lamellae, as confirmed from all unstained control sections. Moreover, apart from autofluorescence, non-specific staining was not seen in any of the immunohistochemical controls.

**Figure 5:**
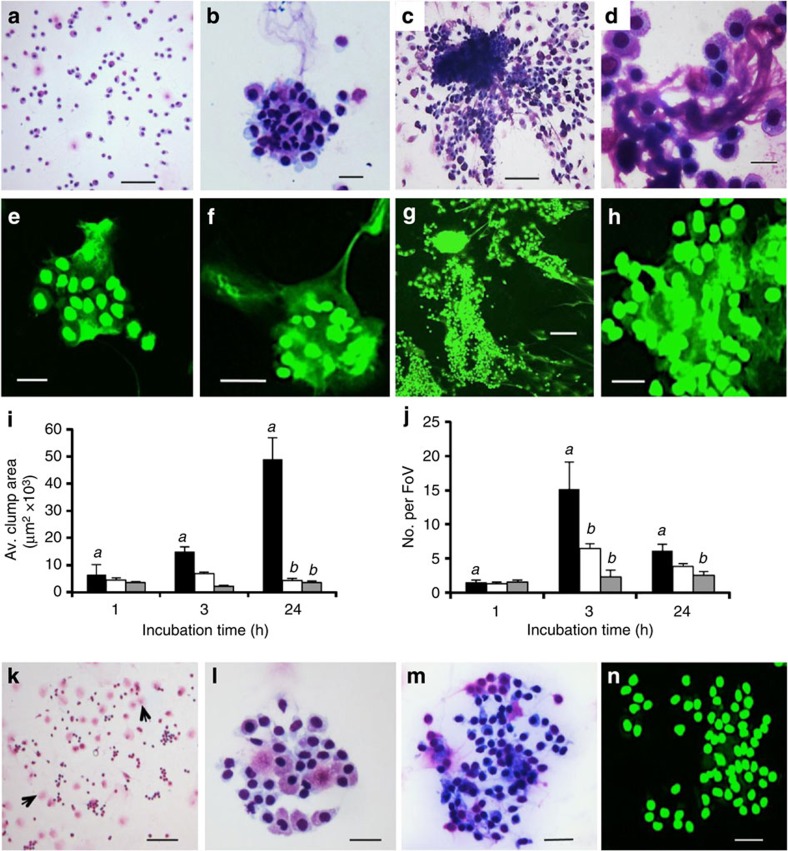
ETosis during the formation of haemocyte clumps in suspension culture. (**a**–**d**) Diff-Quik stained cytocentrifuge preparations of unseparated *C. maenas* haemocytes. (**a**) 24 h, control (no PMA). Scale bar, 100 μm (**b**) 3 h, 0.1 μM PMA. Scale bar, 20 μm. (**c**) 24 h, 0.1 μM PMA. Scale bar, 100 μm. (**d**) Detail of clump (24 h) showing extruded material permeating intact cells. Scale bar, 20 μm. (**e**–**h**) Sytox Green stained cytocentrifuge preparations. (**e**) ETosis in small clump 1 h (0.1 μM PMA). (**f**) Clump showing intact haemocytes entrapped by extracellular chromatin (3 h 0.1 μM PMA). (**g**) 24 h, 0.1 μM PMA. Clump showing strands of chromatin. (**h**) Detail showing extracellular chromatin suffused through the cell matrix (24 h). Scale bars (**e**,**f**,**h**) 20 μm, (**g**) 100 μm. (**i**–**n**) Haemocyte clumps formed in PMA only, PMA+DPI or PMA+DNase. All incubations 10 °C. (**i**,**j**) solid columns=PMA only, open columns=PMA+DNAse-1, grey columns=PMA+DPI. Values are mean±s.e.m. of one representative experiment from three independent experiments. Significant differences between PMA-only treatments over time denoted by *a*. Significant differences between PMA-only and PMA+DNAse or PMA+DPI within one time point indicated by *b*, one-way analysis of variance with Student–Newman–Keuls *post hoc* test. (**i**) Clump size. Values are mean±s.e.m. (*n*=6–52). PMA 1 h versus 24 h, *P*<0.01; PMA 3 h versus 24 h and PMA versus PMA+DNAse-1, both *P*<0.001; PMA versus PMA+DPI *P*<0.05. (**j**) Number of haemocyte clumps (that is, >20 haemocytes in close contact). Values are mean number per field of view (FoV)±s.e.m. (*n*=6). PMA only 1 h versus 24 h, *P*<0.01. PMA only 1 h versus 3 h, and 3 h versus 24 h, both *P*<0.001. PMA versus PMA+DNAse, *P*<0.001; PMA versus PMA+DPI *P*<0.05. (**k**–**m**) Diff-Quik™ stained cytocentrifuge haemocyte preparations. (**k**) 24 h PMA+DPI. Compare with **c**. Arrows indicate degranulated cells. Scale bar, 200 μm. (**l**) Small, loose haemocyte clump, 3 h PMA+DNAse-1. Compare with **b**, **f**. Scale bar, 20 μm. (**m**) Haemocyte clump, 24 h PMA+DNAse-1. Compare with **c**,**d**. Scale bar, 40 μm. (**n**) Haemocyte clump, 24 h PMA+DNAse-1, Sytox Green stain. Compare with **g**,**h**. Scale bar, 20 μm.

To further establish that ETosis plays a key part in the encapsulation response of crabs, additional assays were performed with PMA with or without DPI or DNAse-1. To avoid toxic effects of these reagents within the crab body, an *in vitro* approach was used. Washed unseparated haemocytes in suspension culture in ML-15 medium without these reagents were found to remain unclotted (Fig. 5a), with *ca.* 80% survival over 24 h (Supplementary Fig. 3). However, in the presence of PMA (0.1 μM), distinct haemocyte clumps, resembling capsules, developed over the 24 h incubation period (Fig. 5b–d). Confocal microscopy with Sytox Green staining revealed extracellular chromatin was present within these cell associations (Fig. 5e–h). At 1 h, the clumps were very loose with evidence of externalized chromatin within the haemocyte association (Fig. 5e). At this point, the cell aggregates varied considerably in size and were few in number (Fig. 5i,j). By 3 h, the clumps were larger with extracellular chromatin clearly evident within and around the constituent haemocytes (Fig. 5f), and were significantly more numerous compared with 1 h (*P*<0.001) (Fig. 5j). After 24 h, the clumps contained high numbers of highly compacted haemocytes (Fig. 5g,h). Long strings of haemocytes were seen attaching to some of the complexes (Fig. 5c) and strands of basophilic material were apparent emanating between the attaching and aggregated haemocytes (Fig. 5d). This material is likely to be of nuclear origin as similar strands were discernible on the Sytox Green stained preparations at this time point (Fig. 5g,h). By this time, the clumps were larger, but less numerous compared with those at 3 h (*P*<0.001) (Fig. 5i,j) probably from coalescence of smaller clumps.

Importantly, inclusion of DPI or DNAse-1 in the PMA-stimulated cultures substantially affected the formation of the haemocyte clumps, particularly after 3 and 24 h (Fig. 5k–n). At 1 h, clumps were too few and variable in size to assess statistically, but at 3 h, DPI significantly reduced the number of the clumps compared with the PMA only-treated cultures (*P*<0.001) (Fig. 5j). At 24 h, the clumps were also significantly smaller and fewer in number in the presence of DPI than without it (*P*<0.001; Fig. 5i,j). In the presence of DNAse, the clumps failed to form large compact structures at 3 h (Fig. 5l), and there were significantly fewer than in the PMA-stimulated cultures at this time (*P*<0.001) (Fig. 5j). Those that did form in the presence of DNAse-1 were loosely packed and contained very few strands of basophilic material (Fig. 5l). Crucially at 24 h, the haemocyte aggregates formed in the PMA+DNAse-1 mixtures lacked cohesiveness and evidence of extracellular chromatin (Fig. 5m,n). Neither DPI nor DNAse-1 stimulated haemocyte clumping on their own, although PMA+DPI-treated cultures tended to show more de-granulated cells than those not exposed to this reagent (Fig. 5k). Collectively, these results show that ETosis is involved in the initiation and development of capsule-like cell clumps over 24 h.

### Chromatin release by defence cells from other invertebrates

**Figure 6:**
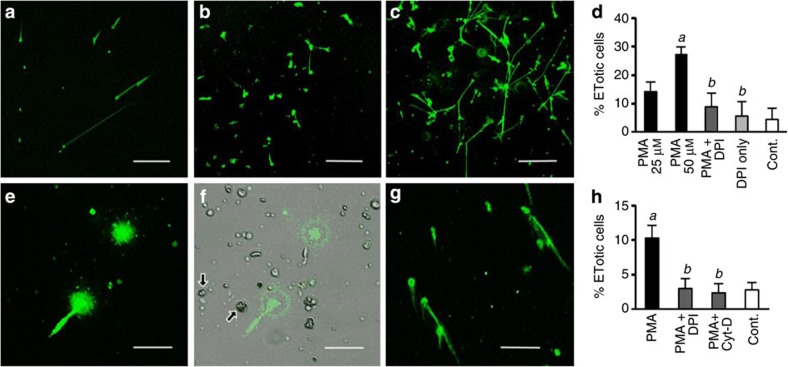
PMA-stimulated chromatin release by defence cells from other invertebrate species *in vitro*. (**a**–**d**) Unfixed *M. edulis* haemocytes in monolayer culture (24–48 h at 10 °C), stained with Sytox Green. (**a**) 0.1 μM PMA, 24 h. (**b**) 25 μM PMA, 48 h. (**c**) 50 μM PMA, 48 h. (**a**–**c**) Scale bars, 300 μm. (**d**) Percentage of ETotic *M. edulis* haemocytes following PMA treatment (25 or 50 μM, 48 h), 50 μM PMA +/− 10 μM DPI or untreated (Cont.). Values means±s.e.m., *n*=3. Significant differences between PMA and untreated haemocytes denoted by *a.* Significant differences between PMA and PMA +/− DPI denoted by *b.* In both cases, *P<*0.05, one-way analysis of variance (ANOVA) with Student–Newman–Keuls (SNK) *post hoc* test. (**e**–**g**) Chromatin release by mesogleal cells isolated from *A. equina* (24 h, 0.1 μM PMA, 10 °C). (**e**) Crude cell extract. (**f**) Merge of **e** with same phase contrast image. Arrows indicate cnidocytes. (**e**,**f**) Scale bars, 33 μm (**g**) Phagocyte-enriched mesogleal extract after trypsin digestion and differential centrifugation. Scale bar, 65 μm. (**h**) Percentage of ETotic cells enriched from the mesoglea of *A. equina* following treatment with PMA (24 h, 0.1 μM), PMA+2 μM DPI, PMA+10 μM cytochalasin D (Cyt-D) or left untreated (Cont.). Values are mean±s.e.m., *n*=3. Significant differences between PMA and untreated cells are denoted by *a.* Significant differences between PMA, and PMA+DPI or PMA+Cyt-D are denoted by *b.* In both cases *P<*0.05, one-way ANOVA with SNK *post hoc* test.

Preliminary *in vitro* experiments were undertaken to assess if haemocytes from a distantly related protostome and an acoelomate invertebrate release chromatin. Using PMA as the stimulus, Sytox Green-positive extracellular material was clearly observed with the mussel, *M. edulis* haemocytes (Fig. 6a–c), although at a lower frequency and only with much higher concentrations of PMA than that of crab HCs over a longer time (Fig. 6d). Only 27.0% (±2.8 s.e.m.) of mussel haemocytes were ETotic, even with a PMA concentration of 50 μM over 48 h. The released material was digested completely by DNAse-1 and as the response is inhibited by DPI (Fig. 6d), we conclude that it is an ETotic phenomenon. Remarkably, cells extracted from the mesoglea of the sea anemone, *A. equina*, also expel DNAse-sensitive nuclear material after 24 h treatment with PMA (0.1 μM) (Fig. 6e–g, Supplementary Movie 2). In crude extracts, the chromatin formed short diffuse structures (Fig. 6e,f) but, after removal of mucus by trypsin digestion, the released chromatin resembled the spread structures similar to those produced by crab and mussel haemocytes (Fig. 6g). The percentage of enriched mesogleal cells showing this response was 10.3% (±1.6% s.e.m.) (Fig. 6h). Pretreatment of the cells with DPI or cytochalasin D significantly reduced this extrusion of chromatin (*P*<0.05) (Fig. 6h) indicating that the process is very likely to be ETosis.

## Discussion

Defence cells from protostome and acoelomate invertebrates can undergo NADPH oxidase or ROS-dependent ETosis, upholding our hypothesis that it represents a primordial cellular phenomenon. We propose that haemocytes that undergo such chromatin release are designated InEPTs (invertebrate extracellular phagocyte traps) to distinguish them from vertebrate ETotic cells. Here, we define a role for InEPTs in host defence of crab, showing induction by bacteria or LPS with the disgorged chromatin able to ensnare microorganisms. Crucially, chromatin released from InEPTs is clearly associated with the formation of haemocyte capsules that are stimulated to develop and lodge in the gill lamellae by injected LPS. Encapsulation is the major process through which bacteria or other non-self materials are cleared from the haemocoel of arthropods^33^,^35^. In *C. maenas*, the phagocytic capability of HCs is relatively low at *ca*. 15–20% (ref. 32) but ~94% of injected bacteria are removed from the circulation within the first hour of infection, mainly to the gill lamellae where they become sequestered in haemocyte capsules^33^,^36^. It is known that in crab, entrapped bacteria are killed within such capsules^37^, no doubt aided by antimicrobial peptides (AMPs) synthesized and stored in the SGCs and GCs^21^,^38^. In the present study, we show that chromatin of InEPTs enable the formation and development of these capsules by binding pathogens and facilitating assembly of other non-ETotic haemocytes to the scaffold. This finding radically changes the present view as to how capsules are initiated in invertebrates^21^ and confirms the original proposal by Brinkmann and Zychlinsky^12^ that chromatin is indeed an evolutionary ancient defence weapon. As revealed by the immunohistochemical analyses presented here, the chromatin aids the aggregation of haemocytes, which become bound to each other, with PXN released from the cells serving to hold the structure firmly together. In addition, the extruded chromatin serves to deliver histones and (likely) other antimicrobial proteins, thereby facilitating the killing of infectious agents caught in the meshes. The externalization of histones on chromatin would further present ‘damage’ signals to the immune system, consistent with the danger model^39^.

A role for extracellular chromatin released by haemocytes actively participating in defence has not previously been demonstrated in any invertebrate. One study on the wax moth, *Galleria mellonella*, reported that injection of purified endogenous nucleic acids simultaneously with pathogenic bacteria prolonged larval survival^40^. The present findings suggest that the injected bacteria were not only killed by AMPs and other immune molecules but also trapped on the externally introduced chromatin and then confined within haemocyte nodules, and thus unable to establish a lethal infection.

As our preliminary experiments found ETotic-style chromatin release similarly occurs in *M. edulis* haemocytes, it is plausible to predict that the process is similarly involved in the development of capsules in bivalves. Certainly, molluscs mount encapsulation reactions to parasites^41^. The less frequent ETotic response by mussel haemocytes and the need for much higher PMA concentrations over a longer incubation time, reported here, is not easily explained, but may relate to the high level of phagocytosis exhibited by bivalves^17^. Phagocytes are closely associated with food transport in these filter feeders so it would be disadvantageous for such animals to disable many haemocytes in this way. Anthozoan cnidarians also display amoebocyte aggregation *in vivo* in response to encounters with fungal hyphae^42^, so it is conceivable that chromatin is involved in this process. Mucus, especially within the mesoglea, probably serves to localize the chromatin and any microorganisms trapped on the meshes. Thus, the spread of invading pathogens or parasites would be stemmed and the infectious material would be brought into intimate contact with antimicrobial agents produced by the tissues or mesogleal cells. AMPs are synthesized by the endoderm of cnidarians^43^,^44^ and amoebocytes of *A. equina* release extracellular ROS^19^ capable of killing bacteria.

As members of the Cnidaria lack a coelom and true circulatory system, chromatin released through ETosis must be an ancient defence process that predates the development of an organized immune system within the mesoderm. In plants, extracellular DNA in root-cap slime of pea, *Pisum sativum*, confers protection against fungal infections^45^, but whether the DNA is extruded in a controlled, NADPH oxidase or ROS-dependent manner, as in vertebrate immune cells, has yet to be established. Moreover, as the chromatin is discharged to the external environment (that is, the soil), rather than within the tissues, it is uncertain if the deployment of chromatin in pea root genuinely represents ETosis. Therefore, at present, it is unknown when ETosis arose in eukaryote evolution, but the present study sheds new light on how early multicellular animals might have protected themselves against prokaryote competitors in their environment.

Our findings also help to inform some of the pathological consequences associated with ETosis in higher vertebrates. We reveal a truly beneficial role for this process in animals without fully tubular vascular systems. For higher vertebrates, however, ETosis may be viewed as a double-edged sword: the trapping and killing of microorganisms is valuable but the ETotic material could become detrimental if it is not resolved or evokes an adaptive response. For instance, while entrapment of haemocytes by externalized chromatin is crucial for capsule development in invertebrates, in mammals, equivalent assembly of leucocytes and platelets on DNA meshes within blood vessels may lead to venous thrombosis^11^,^46^,^47^. Moreover, a recent paper has suggested that NETs can facilitate metastasis of tumours by trapping cancerous cells and enabling their adhesion to healthy tissues^13^. ETosis is further associated with chronic respiratory diseases, such as cystic fibrosis, where the externalized chromatin causes, among other effects, an increase in sputum viscosity that aids microbial colonization and biofilm formation^48^,^49^,^50^.

In mammals, the lack of NETs clearance, together with inappropriate stimulation of an adaptive immune response, may occur in a number of autoimmune diseases; for example, systemic lupus erythematosis^51^ and rheumatoid arthritis^52^. A characteristic of systemic lupus erythematosis is the production of autoantibodies against immunogenic complexes of self-DNA and neutrophil AMPs arising from neutrophil ETosis^51^. Such complexes may pose an additional complication of glomerulonephritis occluding the kidney and impairing renal function^53^. In rheumatoid arthritis, NETs are abundant in the synovial fluid of affected joints and rheumatoid nodules in skin, with autoantibodies sometimes forming against citrullinated vimentin, an intermediate filament protein, externalized on the chromatin^52^. For invertebrates, the problem of autoimmunity is non-existent, as they do not synthesize antibodies and cannot mount antibody-mediated adaptive immune responses. As such, invertebrates facilitate the study of processes central to innate immunity in a non-biased environment (that is, one fully without the presence of clonally derived lymphocytes and their activities).

In conclusion, the InEPTs described here confirm that extracellular chromatin is a primordial defence process that effectively protects against infection and, accordingly, has been conserved through evolution. In higher animals, the advantages of externalized chromatin as an anti-infective strategy remain, but the more complex vasculature of vertebrates and the presence of an adaptive immune system mean that exposed chromatin can be problematic. The phylogenetic history of ETosis as a primitive host defence strategy accounts for its paradoxical effects in higher vertebrates.

## Methods

### Animals and reagents

Specimens were collected from the Forth Estuary, Scotland and kept in seawater (~32‰, 12±2 °C) until use. Only healthy individuals, showing no signs of injury or infection were used. Unless otherwise specified, all reagents were purchased from Sigma Aldrich.

### Preparation of monolayer cultures of crab haemocytes

Haemolymph (2 ml) was withdrawn from the unsclerotized membrane between the merus and the carpus of a cheliped of adult intermolt crabs (8.5±1.0 cm carapace width) into a syringe containing 2 ml of anti-coagulant (0.5 M NaCl; 0.1 M glucose; 30 mM trisodium citrate; 26 mM citric acid (monohydrate); 10 mM ethylene diamine tetra-acetic acid (EDTA); pH 4.6)^16^. The haemocyte populations were separated by isopycnic density centrifugation (25,000*g*, 20 min, 4 °C) on preformed continuous 60% Percoll gradients in 3.2% NaCl^16^. HCs were obtained from the top band, SGCs from the middle band with GCs from the bottom band^16^. Prohaemocytes, normally at very low levels in the haemolymph of crabs, were recruited into the circulation by prebleeding (2 ml) 24 h before collection at the second bleed^54^. They were then isolated by two-step separation, first on 60% Percoll as above and, second on a preformed continuous 40% Percoll gradient in 3.2% NaCl^54^. After washing in 0.22 μm filtered 3.2% NaCl, each of the separated cell populations were re-suspended in modified L-15 medium (ML-15) made by supplementing single-strength medium with 0.4 M NaCl and a penicillin/streptomycin mix (100 U per 100 μg ml^−1^). The haemocytes were cultured in flat-bottomed 24-well plates to allow for cell attachment (1 h, 10 °C) with triplicate cultures of *ca.* 3 × 10^4^ cells made for each animal. A minimum of three animals was used in each experiment.

### Assay of chromatin release by crab haemocytes *in vitro*

ETosis by crab haemocytes was induced with phorbol-12-myristate 13-acetate (PMA) at final concentrations 0.01–1.0 μM. Controls received equal volumes of medium only. The plates were incubated at 10 °C for 1–24 h before staining, unfixed, for 20 min with 1 μM Sytox Green (Invitrogen) and visualization with a Leica (DMIRE2) TCS2 confocal microscope (excitation 504 nm; emission 523 nm). Using a combination of fluorescence and phase contrast microscopy, the percentages of viable, chromatin extruding or non-ETotic dead cells were calculated from a minimum of 300 cells from a minimum of three fields of view from replicate experiments using the image analysis software, ImageJ (National Institutes of Health, USA). In judging ETotic cells, no distinction was made between diffuse or spread forms of chromatin. To confirm that the released material was of nuclear origin, DNAse-1 (Fermentek) (100 U ml^−1^ final concentration) was added to HC cultures that been treated with 0.1 μM PMA 24 h previously and then stained, unfixed, with Sytox Green (20 min). The cultures were incubated (10 °C) with changes recorded by live time-lapse photo-microscopy.

### Chromatin decoration *in vitro*

Immunocytochemistry was used to investigate decoration of the chromatin from crab HCs with H2A and PXN. The primary antibodies were mouse anti-H2A IgG1 (New England Biolabs) or rabbit anti-human myeloperoxidase IgG (Dako), an antibody that recognizes PXN, both at a dilution of 1:200 in phosphate-buffered saline (PBS) supplemented with 3% bovine serum albumin (BSA). The secondary antibodies were Alexa Fluor 594F(ab)_2_ goat anti-mouse IgG (excitation 590 nm; emission 617 nm) or Alexa Fluor 488 goat anti-rabbit IgG (excitation 495 nm; emission 519 nm) (both from Invitrogen) to detect H2A and PXN, respectively. Both secondary antibodies were diluted 1:300 in PBS supplemented with 3% BSA.

Monolayer cultures of HCs were stimulated with 0.1 μM PMA, as above, except that the cells were cultured on glass coverslips laid in the plate wells, rather than directly on the well surface. After 24 h, the culture medium was removed and the cells fixed with 4% paraformaldehyde in 2% NaCl (30 min). The cells were washed three times with PBS, permeabilized with 0.1% Triton X-100 (5 min) and washed thrice again with PBS before blocking in 3% BSA v/v in PBS for 1 h. The haemocytes were then incubated for 1.5 h with the primary antibodies and, after further three washes in PBS, the slides were incubated (1.5 h) with the secondary antibodies. Controls included haemocytes without primary and secondary antibodies, haemocytes incubated only with secondary antibodies and haemocytes treated with mouse or rabbit sera instead of primary antibodies. For each control, 3% BSA in PBS was substituted for the relevant antibody solution(s). DNA and actin were localized, respectively, with 5 μM Draq 5 (excitation 647 nm; emission 681 nm) (Biostatus) or 0.15 μM rhodamine phalloidin (excitation 540 nm; emission 565 nm) (Invitrogen) plus the secondary antibodies. The haemocytes were finally washed three times with PBS and mounted onto sterile glass slides with Vectashield (Vector Laboratories) before examination by confocal microscopy, as above.

### Electron microscopy

Material was fixed (2 h) in 2.5% glutaraldehyde, in 0.1 M sodium cacodylate (CAC), pH 7.2 then washed in PBS and dehydrated through a graded series of ethanols. For SEM, the material was critical point dried (3 h), sputter coated with gold and visualized with a Quanta 3D FEG–FEI microscope (FEI UK). For transmission electron microscopy, fixed samples were washed in three changes of CAC, post fixed in 1% osmium tetroxide, in 0.1 M CAC, for 45 min and washed three times, as above, before dehydration in 50, 70, 90 and 100% normal grade acetone (10 min each). This was followed by two 10 min washes in Analar acetone and embedding in araldite. Ultrathin sections (60 nm thickness) were stained with uranyl acetate and lead citrate and viewed using a Phillips CM120 microscope (FEI UK). The diameters of globular and smooth domains of extracellular chromatin released from crab HCs were measured from high resolution SEM using ImageJ. The diameters of ~15 randomly selected domains were measured and the average diameter calculated (nm).

### Inhibition of ETosis by crab HCs *in vitro*

Fresh HC monolayer cultures were incubated with either 2 μM diphenyleneiodonium, 50 μM apocynin (1-(4-hydroxy-3-methoxyphenyl) ethanone) (Calbiochem), 1 μM Ro-31-8220 (3-(3-(4-(1-methyl-1*H*-indol-3-yl)-2,5-dioxo-2,5-dihydro-1*H*-pyrrol-3-yl)-1*H*-indol-1-yl)propyl carbamimidothioate) or 10 μM cytochalasin D (all for 1 h, 10 °C) before addition of PMA, as above. Controls consisted of stimulated haemocytes minus inhibitor or non-stimulated haemocytes plus inhibitor. Chromatin release was detected and quantified as above.

### Stimulation of ETosis with LPS or bacteria *in vitro*

Cultures of crab HCs were set up, as above, but treated with LPS from *Escherichia coli* serotype 0111:B4 (final concentration 0.1 μg ml^−1^) or a washed suspension of the fish pathogen, *Listonella anguillarum* (ATCC 43305), (3 × 10^5^ ml^−1^ final) instead of PMA. Controls received medium only. All cultures were incubated for 24 h (10 °C) then processed and visualized as above. Inhibition experiments were also performed with DPI, as above.

### ETosis in the host defences of *C. maenas in vivo*

To ascertain if ETosis or released chromatin participates in encapsulation reactions, crabs were given injections of LPS (100 μl of 20 μg ml^−1^ LPS from *Escherichia coli* serotype 0111:B4 in sterile 3.2% NaCl). The injections were made through the unsclerotized membrane between the carapace and the coxa of the fourth pereiopod (walking leg)^33^. The dose given represents a final concentration of ~0.1 μg ml^−1^ within the haemocoel and mimics infection without the risk of pathology. It stimulates the formation of haemocyte capsules in the gills, the main site for the sequestration of non-self agents^33^,^36^. Control crabs were given 100 μl of 0.22 μm filtered sterile 3.2% NaCl instead of LPS. Both treatment groups were housed separately in the aquarium until sampling at 1, 3 or 24 h when they were killed. The gills were excised and fixed in 2.5% glutaraldehyde before processing and embedding in paraffin wax. Sections (4-μm thick) were cut and stained with 300 nM 2-(4-amidinophenyl)-1H-indole-6-carboxamidine (DAPI) (excitation 375 nm; emission 461 nm) (Invitrogen) or haemotoxylin and eosin and examined by confocal microscopy, as above, or with a Zeiss AxioSkop fluorescence microscope with QCapturePro software.

Immunohistochemistry was used to demonstrate the role of externalized chromatin in encapsulation reactions *in vivo*. The primary antibodies were the same as those used to study chromatin decoration *in vitro* and at the same dilution, described above. To induce the haemocytes to form capsules, crabs were given injections of LPS or, for controls, 3.2% NaCl, and processed as above except that gill tissue was fixed in paraformaldehyde plus 2% NaCl before embedding and sectioning. All sections were incubated for 30 min with 1 mg ml^−1^ sodium borohydride to reduce autofluorescence then washed three times in PBS for 5 min per wash. A blocking solution of 3% BSA in PBS was added to the sections for 1 h before overnight incubation at 4 °C with the primary antibodies as above. Sections were then washed three times and incubated for 2 h in the dark (4 °C) with secondary antibodies, as above. Sections were washed again three times and incubated for a further 1 h with 5 μM TO-PRO-3 iodide (excitation 642 nm; emission 661 nm) (Invitrogen) to stain both extracellular and intracellular DNA. Finally, sections were washed three times, submerged for 5 s in Millipore water and then mounted using ProLong Gold anti-fade mounting medium (Invitrogen). Immunohistochemical controls paralleled those specified for the chromatin decoration *in vitro* above. Slides were examined using a confocal microscope as above.

### ETosis by crab haemocytes in suspension culture

Unseparated haemocytes diluted in anticoagulant (above) were centrifuged (500*g*, 5 min, 4 °C) and re-suspended in ML-15 in sterile 15 ml tubes to give a concentration of 1 × 10^6 ^ml^−1^. The experimental tubes received PMA (final concentration 0.1 μM) while the controls were given the same volume of ML-15. All tubes were incubated at 10 °C with subsamples (200 μl) aspirated, after gentle mixing, at 1, 3 or 24 h for cytocentrifugation in a Shandon Cytospin 3 at 7*g* (250 r.p.m.) for 3 min. Two sets of slides were prepared for each time and each treatment. One was air-dried, fixed with methanol (1 min) and stained with Diff-Quik (Gamidor) (red, then blue: 1.5 min each), before air-drying, mounting in DePex (30 min) and examination by light microscopy as above. The other was fixed in 2.5% glutaraldehyde in 3.2% NaCl, stained with 1 μM Sytox Green (20 min) and examined with the Zeiss AxioSkop microscope as above.

To confirm the role of chromatin in encapsulation *in vitro*, further sets of cultures were set up in which the haemocytes were pre-incubated with either DPI (4 μM for 1 h) or DNAse-1 (200 u ml^−1^ for 15 min) before the addition of PMA, as above. Higher concentrations of DPI and DNAse-1 were used here because of the greater number of (unseparated) haemocytes present in the suspension cultures compared to those in the single-population monolayer cultures. Controls received DPI or DNAse-1, but were given 3.2% NaCl instead of PMA. The tubes were then incubated, sampled and processed for light microscopy, as above. To assess clump size and frequency, images were recorded as above. To make a clear distinction between controls and treated cells, clumps were defined as a closely packed aggregation of 20 or more cells. The image analysis software ImageJ was used to measure clump numbers and areas (μm^2^) from a minimum of six fields of view (× 100 magnification) per experiment. The experiment was repeated three times.

### Chromatin release by defence cells from other invertebrates

Mussel haemolymph (0.4 ml) was withdrawn from the adductor muscle of each animal into a syringe containing an equal volume of 0.05 M Tris–HCl buffer, pH 7.6, supplemented with 2% glucose, 2% NaCl and 0.5% EDTA^17^. The haemocytes were counted immediately and 3 × 10^4^ ml^−1^ were seeded into wells of a plastic 24-well plate, as above. Mesogleal cells were extracted from *A. equina* by excising the mesenteric filaments, mixing them with an equal volume of homologous fluid drained from the coelenteron and then vigorously pipetted to dissociate the amoebocytes^19^. Digests were performed with 0.01% trypsin in 0.4 M NaCl (10 min, 10 °C), centrifuged (300*g*, 5 min, 10 °C) and the supernatant re-centrifuged as before. Panning was used to further enrich the phagocytes leaving *ca.* 1 × 10^4^ ml^−1^ per well. Cells from both species were incubated at 10 °C and then assayed for chromatin release by confocal microscopy following PMA treatment and staining with Sytox Green, as above.

### Statistical analysis

Data were tested for normality using the Kolmogorov–Smirnov test and tested for significance using one-way analysis of variance with a Student–Newman–Keuls *post hoc* test. Where appropriate, data were log or arc sine transformed before analysis. Probability values of *P*<0.05 were regarded as statistically significant.

## Author contributions

V.J.S. conceived the project and designed the experiments with E.A.D., A.G.R. and C.T.R. The experimental work was performed primarily by C.T.R. Advice, intellectual input, facilities and reagents were provided by V.J.S., E.A.D., A.G.R. and R.D.G. The data were analyzed by E.A.D., C.T.R. and V.J.S. V.J.S. wrote the paper and all authors contributed equally to its revision.

## Additional information

**How to cite this article:** Robb, C. T. *et al*. Invertebrate extracellular phagocyte traps show that chromatin is an ancient defence weapon. *Nat. Commun.* 5:4627 doi: 10.1038/ncomms5627 (2014).

## Supplementary Material

Supplementary Information Supplementary Figures 1-3 and Supplementary Reference

Supplementary Movie 1 DNAse digestion of extracellular chromatin released from crab hyaline cells in vitro. Confocal time-lapse video of DNAse digestion of chromatin extruded from C. maenas HCs in vitro over 280 min. Haemocytes were cultured in ML-15 medium with 0.1 μM PMA (24 h) to stimulate extracellular traps then 100 U ml-1 DNAse was added. Scale bar = 300 μm.

Supplementary Movie 2 DNAse digestion of extracellular chromatin released by mesogleal cells of sea anemone. Confocal time-lapse video of DNAse digestion of extruded chromatin from enriched cultures of cells from the mesenteric filaments of A. equina in vitro over an 80 min period. The cells were cultured in ML-15 with 0.1 μM PMA (24 h, 10 oC) to stimulate extracellular traps before addition of 100 U ml-1 DNAse-1. Scale bar = 55 μm.
