# Supplementary material for: Invertebrate extracellular phagocyte traps show that chromatin is an ancient defence weapon
Source: Nat Commun. 2014 Aug 13;5:4627. doi: 10.1038/ncomms5627 (PMC4143918; doi:10.1038/ncomms5627)
Supplement: Supplementary Information — Supplementary Figures 1-3 and Supplementary Reference [file ncomms5627-s1.pdf]

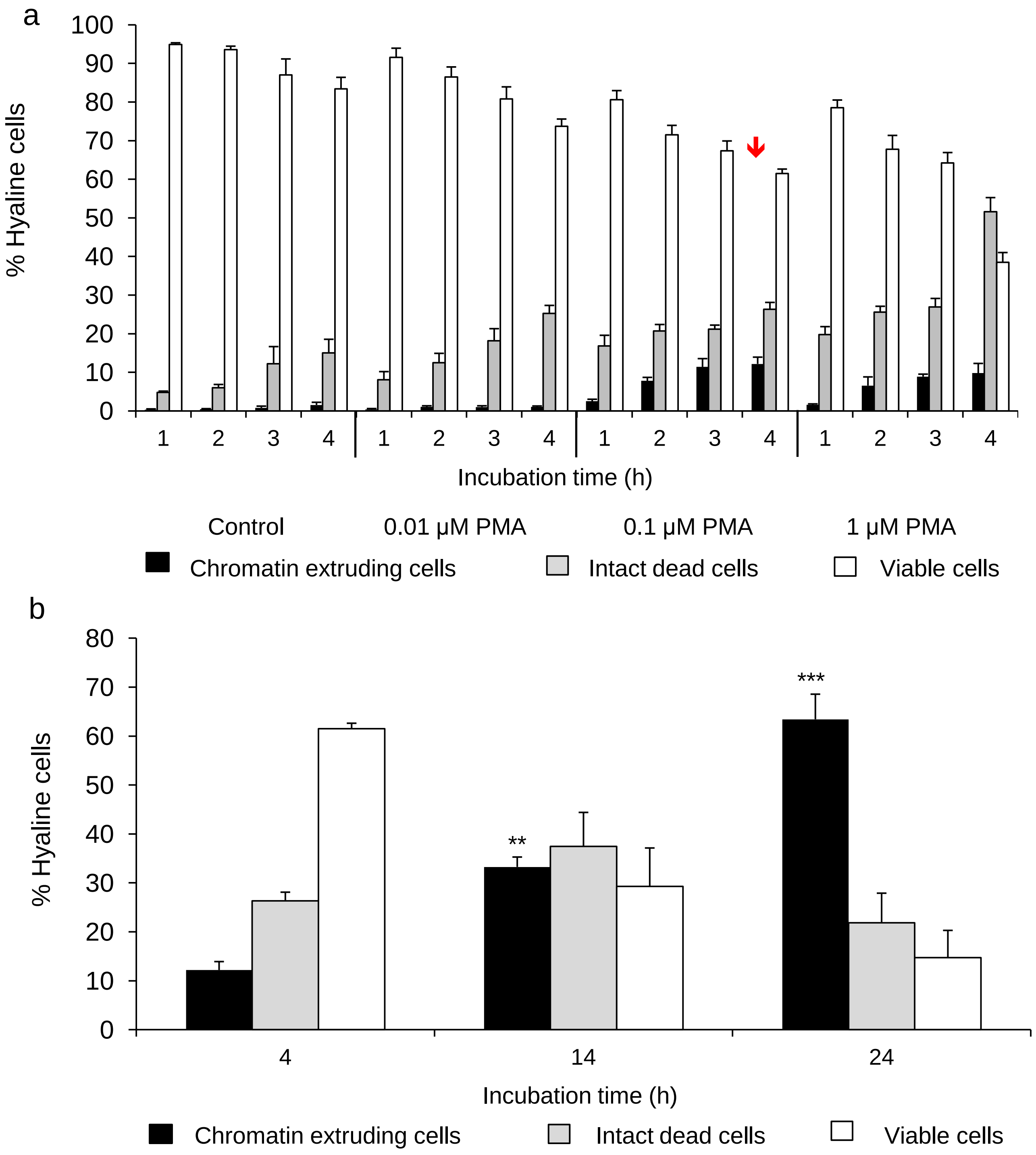

Supplementary Figure 1. Effect of PMA on separated hyaline cells (HC) from crab *in vitro.* Concentration-time analysis of the effect of PMA on separated HCs from *C. maenas* cultured in ML-15 medium. **(a)** Percentage of chromatin-extruding haemocytes with different concentrations of PMA over 4 h, as determined by confocal microscopy. Arrow indicates the optimal concentration (0.1 µM) of PMA for chromatin extrusion combined with overall lower value of non-ETotic cell death. **(b)** Percentage of chromatin-extruding cells over 24 h with 0.1 µM PMA, showing significant differences (one-way ANOVA with Student-Newman-Keuls *post hoc* test) between chromatin-extruding haemocytes at 14 and 24 h compared to 4 h. ** represents $P<0.01$; *** represents $P<0.001$. Values for (a) and (b) are means ± s.e.m. (n = 3).

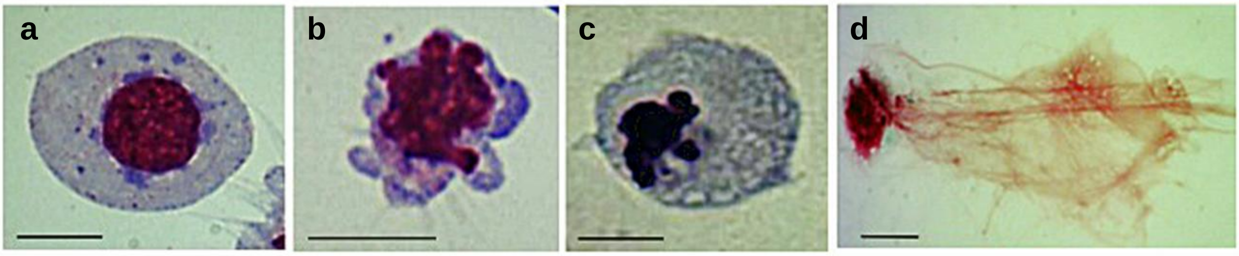

Supplementary Figure 2. Morphological appearance of crab hyaline cells during apoptosis compared to ETosis as revealed by cytocentrifugation. (a) Control, viable haemocyte with intact plasma and nuclear membranes. (b-c) Apoptotic HCs cultured for 24 h at 10 ^{o}C in ML-15 medium containing 10 μM of the cyclin-dependent-kinase inhibitor, R-roscovitine (Calbiochem), an inducer of apoptosis[1] **(b)** Membrane blebbing **(c)** Condensed nucleus showing karyorrhexis. **(d)** ETotic cell with extruded chromatin after treatment with 0.1 μM PMA (24 h). All cytocentrifuge preparations were stained with DiffQuik™. Scale bars (a-c) 10 μm, (d) 20 μm.

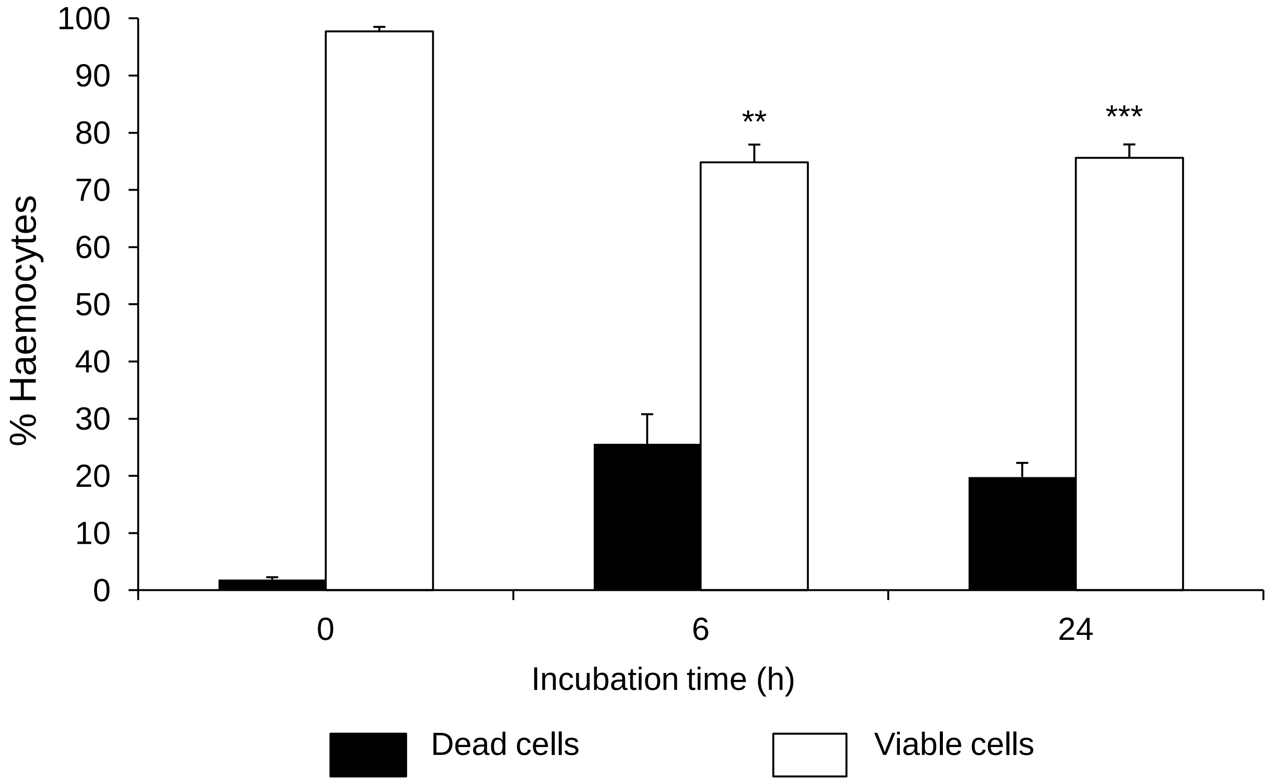

Supplementary Figure 3. Viability of *C. maenas* haemocytes in suspension culture. Washed unseparated *C. maenas* haemocytes cultured in suspension in ML-15 medium at 10 °C and analysed by flow cytometry at 0, 6 and 24 h. The haemocytes were stained with bovine lactadherin-FITC and propidium iodide. *** $P<0.001$ viable versus dead cells at 24 h, ** $P<0.01$ viable versus dead cells at 6 h. Significance calculated by one-way ANOVA with a Student-Neumann-Keuls *post-hoc* test. Values are means ± s.e.m. ($n = 3$).

Supplementary reference

1. Rossi, A.G. *et al*, Cyclin-dependent kinase inhibitors enhance the resolution of inflammation by promoting inflammatory cell apoptosis. *Nat. Med.* **12**, 1056-1064 (2006).
